# Strain analysis in CRT candidates using the novel segment length in cine (SLICE) post-processing technique on standard CMR cine images

**DOI:** 10.1007/s00330-017-4890-0

**Published:** 2017-06-27

**Authors:** Alwin Zweerink, Cornelis P. Allaart, Joost P. A. Kuijer, LiNa Wu, Aernout M. Beek, Peter M. van de Ven, Mathias Meine, Pierre Croisille, Patrick Clarysse, Albert C. van Rossum, Robin Nijveldt

**Affiliations:** 1 Department of Cardiology, and Institute for Cardiovascular Research (ICaR-VU), VU University Medical Center, Amsterdam, The Netherlands; 2 Department of Physics and Medical Technology, VU University Medical Center, Amsterdam, The Netherlands; 3 Department of Epidemiology and Biostatistics, VU University Medical Center, Amsterdam, The Netherlands; 4 Department of Cardiology, University Medical Center, Utrecht, The Netherlands; 5 Univ Lyon, UJM-Saint-Etienne, INSA, CNRS UMR 5520, INSERM U1206, CREATIS, F-42023 Saint-Etienne, France

**Keywords:** Cardiovascular magnetic resonance (CMR), Segment length in cine (SLICE) technique, Myocardial tagging (CMR-TAG), Myocardial strain analysis, Cardiac Resynchronization Therapy (CRT)

## Abstract

**Objectives:**

Although myocardial strain analysis is a potential tool to improve patient selection for cardiac resynchronization therapy (CRT), there is currently no validated clinical approach to derive segmental strains. We evaluated the novel segment length in cine (SLICE) technique to derive segmental strains from standard cardiovascular MR (CMR) cine images in CRT candidates.

**Methods:**

Twenty-seven patients with left bundle branch block underwent CMR examination including cine imaging and myocardial tagging (CMR-TAG). SLICE was performed by measuring segment length between anatomical landmarks throughout all phases on short-axis cines. This measure of frame-to-frame segment length change was compared to CMR-TAG circumferential strain measurements. Subsequently, conventional markers of CRT response were calculated.

**Results:**

Segmental strains showed good to excellent agreement between SLICE and CMR-TAG (septum strain, intraclass correlation coefficient (ICC) 0.76; lateral wall strain, ICC 0.66). Conventional markers of CRT response also showed close agreement between both methods (ICC 0.61–0.78). Reproducibility of SLICE was excellent for intra-observer testing (all ICC ≥0.76) and good for interobserver testing (all ICC ≥0.61).

**Conclusions:**

The novel SLICE post-processing technique on standard CMR cine images offers both accurate and robust segmental strain measures compared to the ‘gold standard’ CMR-TAG technique, and has the advantage of being widely available.

***Key Points*:**

*• Myocardial strain analysis could potentially improve patient selection for CRT.*

*• Currently a well validated clinical approach to derive segmental strains is lacking.*

*• The novel SLICE technique derives segmental strains from standard CMR cine images.*

*• SLICE-derived strain markers of CRT response showed close agreement with CMR-TAG.*

*• Future studies will focus on the prognostic value of SLICE in CRT candidates.*

**Electronic supplementary material:**

The online version of this article (doi:10.1007/s00330-017-4890-0) contains supplementary material, which is available to authorized users.

## Introduction

Myocardial strain analysis plays a key role in the quantitative assessment of global left ventricular (LV) function in various cardiac pathologies and provides prognostic information over conventional parameters [1]. Heart failure (HF) patients with left bundle branch block (LBBB) show regional timing differences causing an imbalanced contractile function throughout the LV and, in particular, across the septal and lateral wall [2–4]. Segmental strains can be used to calculate parameters of mechanical discoordination (opposing shortening and stretching within the LV). This paradoxical wall deformation underlies an inefficient pump function and is considered to be the functional substrate amenable to resynchronization. Therefore, segmental strain measurements are increasingly recognized for their additional value in the selection of HF patients for cardiac resynchronization therapy (CRT) [4–7].

Echocardiographic techniques, such as the speckle tracking method, provide strain measures on a global and segmental scale. Although echocardiography is widely available, strain analysis is highly dependent on the image quality provided by the acoustic window and remains limited because of its reproducibility [8, 9]. In the meantime, cardiovascular magnetic resonance (CMR) has rapidly emerged as a robust imaging modality with high accuracy to provide detailed information on cardiac morphology, function and tissue characterisation [10]. In particular, CMR myocardial tissue tagging (CMR-TAG) produces high quality strain measures on a global and segmental level. It is considered to be the gold standard, but is not widely available and is predominantly used for scientific purposes [11]. On the other hand, CMR cine imaging is part of every standard clinical protocol and is increasingly utilized in the screening of CRT candidates to determine the LV ejection fraction. Therefore, CMR feature-tracking (CMR-FT) post-processing techniques have been developed to derive strain measurements from standard cine images [12]. Although global strain measures show reasonable agreement with the CMR-TAG technique, segmental strain measures have repeatedly been proven to be insufficient [13–17]. This study evaluates the novel segment length in cine (SLICE) post-processing technique on standard CMR cine images to derive accurate segmental strain measures in CRT candidates without the use of commercial software.

## Methods

### Study population

A subset of 27 patients who underwent CMR examination including CMR-TAG was selected from the *Markers And Response to CRT* (MARC) study [18]. This multi-centre, prospective, non-randomized study was designed to investigate the relationship of a set of (bio) markers to predict response to CRT. Inclusion and exclusion criteria are reported in the supplemental material. All subjects gave written informed consent and the local medical ethics committee (VU University Medical Center, Amsterdam) approved data collection and management. The investigation conforms with the principles outlined in the Declaration of Helsinki.

### Image acquisition

CMR imaging was performed on a 1.5 T whole body system (Magnetom Avanto, Siemens, Erlangen, Germany) with the use of a phased array cardiac receiver coil. Both CMR cine images and CMR-TAG images were obtained in the same examination. Typical image acquisition parameters are given in the Supplemental Material. Standard CMR cine images were acquired using a retrospectively ECG-gated balanced steady-state free-precession (SSFP) sequence during end-expiratory breath holding. A stack of 8–12 consecutive short axis cine images was acquired covering the full LV. Subsequently, high temporal resolution cine imaging of the LV in the three-chamber view was performed to assess the opening and closure times of the mitral and aortic valve. CMR-TAG images were acquired at three short-axis slices (basal, mid, apical) using a complementary spatial modulation of magnetization (CSPAMM) line tagging sequence with segmented ECG-gated acquisitions and serial breath holds [19]. Myocardial scar territory was assessed by late gadolinium enhancement imaging, and infarct size was measured using the full width at half maximum method [20].

### Post-processing images using the segment length in cine (SLICE) technique

**Fig. 1:**
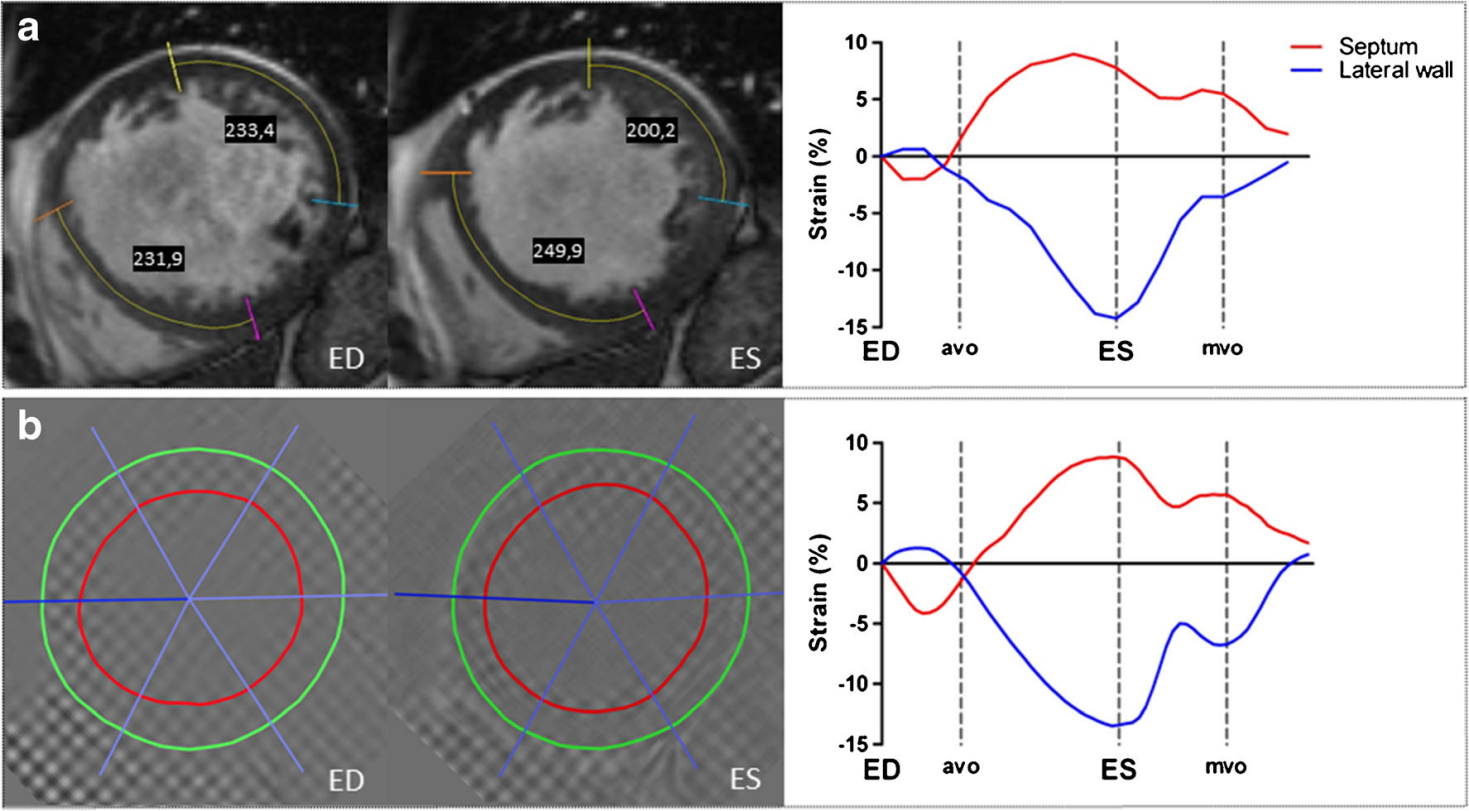
Segmental strain analysis by the segment length in cine (SLICE) method and the ‘gold standard’ cardiovascular magnetic resonance – myocardial tissue tagging (CMR-TAG) technique. A typical example of a left bundle branch block (LBBB) patient with strain analysis using the SLICE technique on short axis cine images (**a**) and using the ‘gold standard’ CMR-TAG technique (**b**). On the cine images, the lateral wall segment shortens from 233.4 to 200.2 pixels corresponding with the −14.2% systolic strain, whereas the septum lengthens from 231.9 to 249.9 pixels corresponding with the +7.7% systolic strain. These systolic (ES) strain values are displayed in the right diagram (**a**). Automated strain analysis of the tagged images shows similar results in this patient (**b**). *ED* end-diastole, *avo* aortic valve opening, *ES* end-systole (by aortic valve closure), *mvo* mitral valve opening

The SLICE analysis of standard cine images was performed by two post-processing steps as illustrated in Fig. S1 in the Supplemental Material. First, the slice position with short-axis cine images, corresponding with the mid-LV slice-location of CMR-TAG images, was selected in *Qmass* (v7.6, Medis, Leiden, The Netherlands). Two endocardial anatomical landmarks (trabeculae) delimiting the septum were chosen in the end-diastolic frame and checked for traceability throughout the cardiac cycle (i.e. limited through-plane motion). Marks were placed perpendicular to the myocardium throughout all phases. This procedure was repeated for the lateral wall segment. Subsequently, marked cine images were exported to *ImageJ* [21] for the second processing step. Segment length of the septum was manually measured between both marks, over the midline of the myocardium in each phase, and expressed as a percentage of the end-diastolic segment length (Fig. 1a). This procedure was repeated for the lateral wall segment. This measure of frame-to-frame segment length change was compared to CMR-TAG circumferential strain measurements in a similar slice position (Fig. 1b), as described below.

### Post-processing myocardial tissue tagging (CMR-TAG)

The CMR-TAG analysis was performed by dedicated software using the SinMod technique (*inTag,* CREATIS, Lyon, France) [22]. The software runs as a plug-in for OsiriX (v6.5, Pixmeo, Switzerland). The semi-automated analysis is described in the Supplemental Material.

### Measures of basic strains, mechanical dyssynchrony and discoordination

**Fig. 2:**
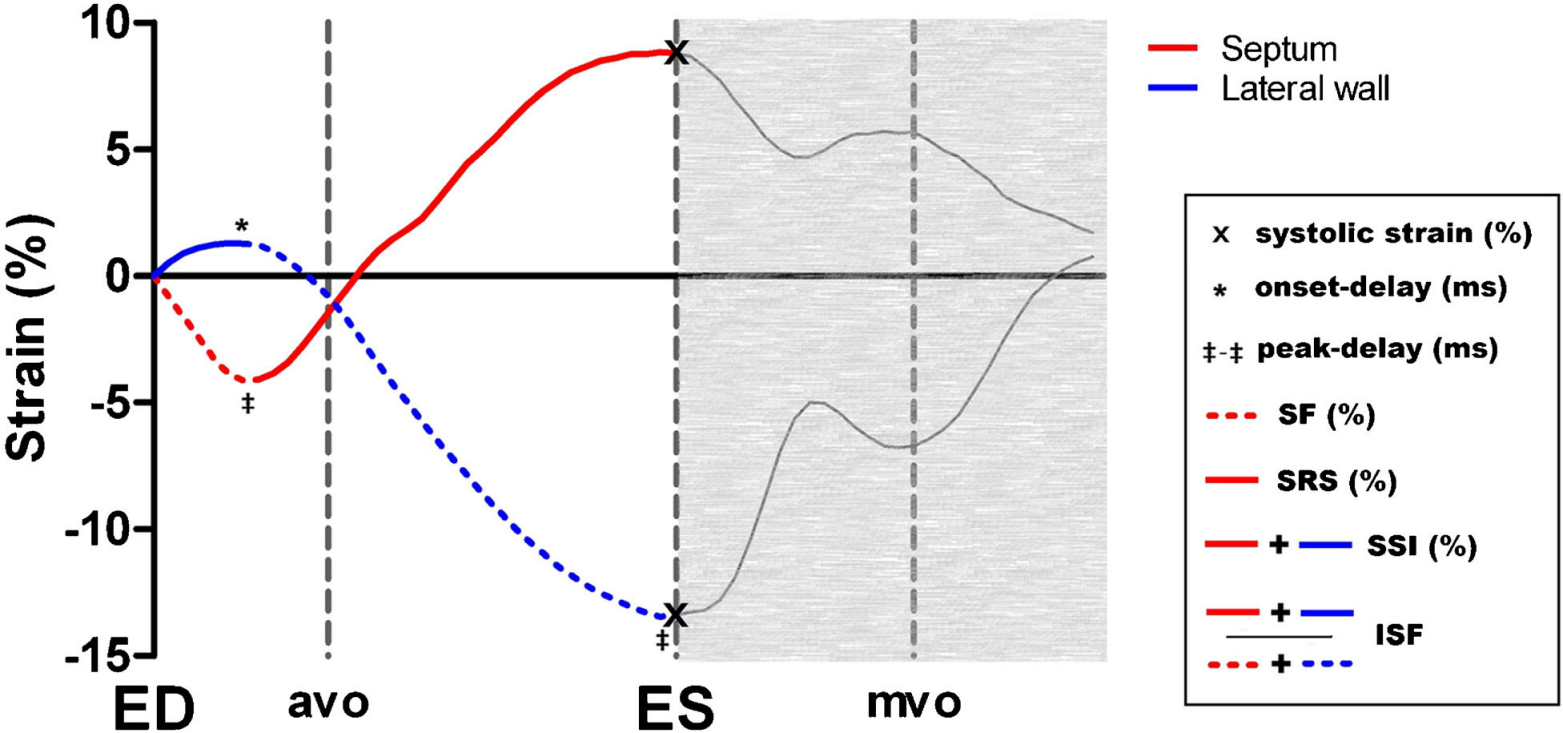
Measures of mechanical dyssynchrony and discoordination. This diagram displays regional systolic strains and time-based parameters of mechanical dyssynchrony, including septum to lateral delay in onset contraction (onset-delay) and difference in time to peak strain between the septum and lateral wall (peak-delay). Strain-based parameters of discoordination are septal flash (SF), systolic rebound stretch of the septum (SRS), systolic stretch index (SSI) and internal stretch index (ISF). *ED* end-diastole, *avo* aortic valve opening, *ES* end-systole (by aortic valve closure), *mvo* mitral valve opening

**Fig. 3:**
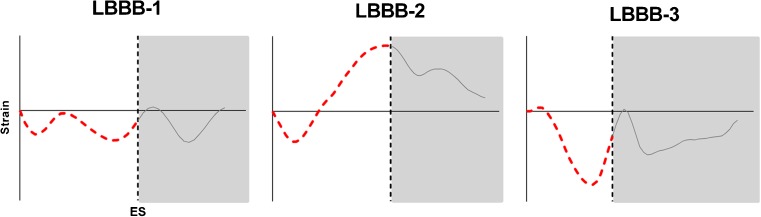
Pre-specified septum strain patterns. Septum strain patterns are classified as double peaked shortening (LBBB-1); predominant stretching (LBBB-2) or pseudonormal shortening (LBBB-3). *ES* end-systole (by aortic valve closure)

Four subsets of strain parameters were evaluated including (i) basic strains, (ii) mechanical dyssynchrony, (iii) discoordination and (iv) pre-specified septal strain patterns. (i) Systolic strain was calculated as the percentage change in segment length at end-systole in comparison to end-diastole. (ii) Time-based dyssynchrony parameters were defined by the delay in onset contraction between the septal and lateral wall (onset-delay) and the time difference between peak shortening (peak-delay) [5]. (iii) Strain-based discoordination markers were assessed by the following commonly used markers: systolic rebound stretch of the septum (SRS) was defined as the cumulative amount of systolic stretch after initial shortening [5]; SRS was combined with stretch of the lateral wall to calculate the systolic stretch index (SSI) [23]; the internal stretch factor (ISF) was calculated by dividing the total amount of systolic stretch (SSI) by the total amount of systolic shortening in both regions [4]. Furthermore, septal flash (SF) was measured as the amount of shortening of the septum before stretching during the isovolumetric contraction phase [24]. (iv) Septal strain patterns were classified as: double-peaked systolic shortening (LBBB-1); early pre-ejection shortening followed by prominent systolic stretch (LBBB-2); and pseudonormal shortening with a late-systolic shortening peak and less pronounced end-systolic stretch (LBBB-3) [25]. Figure 2 illustrates the assessment of dyssynchrony and discoordination parameters and Fig. 3 shows the pre-specified septal strain patterns.

### Statistical analyses

The commercially available Statistical Package for Social Sciences software (IBM SPSS Statistics for Windows, Version 20.0. Armonk, NY, USA) was used for statistical analysis. Continuous variables are expressed as mean±standard deviation. Non-parametric data are expressed as median (25–75% interquartile range). Categorical variables are presented as absolute numbers and percentages. Agreement between strain values measured by the SLICE and CMR-TAG method was assessed using a two-way mixed-effect intraclass correlation coefficient for absolute agreement (ICC) and Bland-Altman analysis. Intra- and interobserver variabilities are expressed using two-way random ICCs. Firstly, the primary executive investigator independently analysed all images. Secondly, 15 randomly selected patients were re-analysed by the same investigator to assess intra-observer agreement. Lastly, a second reader analysed the same subset of patients for interobserver agreement. Analysis per reader was strictly separated. Agreement was considered excellent (ICC ≥0.75), good (ICC 0.6–0.74), moderate (ICC 0.4–0.59) or poor (ICC <0.40) [26]. Agreement of septal strain patterns assessed by SLICE and CMR-TAG was tested by Cohen’s Kappa coefficient, using the following scale: excellent (k ≥0.81), good (k 0.61–0.8), moderate (k 0.41–0.60) or poor (k <0.40).[27] Student’s t-test (independent or paired) or non-parametric test was used to compare groups when appropriate. Correlations were assessed using Pearson’s correlation coefficient or, when normal distribution was absent, Spearman’s Rho correlation coefficient. A *p*-value of <0.05 was considered statistically significant.

## Results

Twenty-seven CRT candidates (age 65±10 years, 16 men) were included in this study. Baseline patient characteristics are listed in Table 1. From a total of 54 CMR-TAG septum segments, 48 (89%) were considered analysable. For the lateral wall, this was 49 (91%) of the 54 segments, resulting in a total of 97 (90%) out of the 108 CMR-TAG segments. For the SLICE analysis method, all 27 (100%) septum and all 27 (100%) lateral wall segments were analysable.

**Table 1:** Patient characteristics

Parameter	N = 27
Clinical	
Age (years)	65 ± 10
Sex (male/female)	16/11
Aetiology (ICMP/NICMP)	7/20
QRS duration (ms)	183 (167–194)
CMR	
Indexed EDV (m/m^2^)	145 (135–183)
Indexed ESV (ml/m^2^)	100 (90–151)
Indexed LV mass (g/m^2^)	68 (59–85)
EF (%)	27 ± 9
CO (L/min)	5.6 ± 1.6
Scar mass ICMP (% LV mass), N = 7	15 (5–20)

*CMR* cardiovascular magnetic resonance *ICMP* ischaemic cardiomyopathy, *NICMP* non-ischaemic cardiomyopathy, *EDV* end-diastolic volume, *ESV* end-systolic volume, *EF* ejection fraction, *CO* cardiac output

### Segmental strain measures by SLICE and CMR-TAG

Measures of segmental strain, dyssynchrony and discoordination analysed by the SLICE and CMR-TAG techniques are given in Table 2. Comparing segmental strains within the same patient using the ‘reference’ CMR-TAG technique showed significantly more negative systolic values in the lateral wall compared to the septum (−12.5±3.5% vs. +3.5±6.4%; *p*<0.001). The lateral wall generally followed a normal contraction pattern with a relatively small variation in systolic strain (range −16.2% to −2.0%) while the septum often demonstrated an opposite strain pattern with a large variation in systolic strain among patients (range −8.4% to +14.3%). Using the SLICE technique, segmental systolic strain was also found to be significantly more negative in the lateral wall compared to the septum (−13.9±4.0% vs. +1.0±6.4%; *p*<0.001). Variation in systolic strain was comparably less in the lateral wall (range −21.0% to −7.5%) than in the septum (−12.5% to +9.5%). Comparing absolute strain values between the SLICE and the CMR-TAG technique, more negative systolic strain values were found both in the lateral wall and in the septum using the former analysis (−13.9±4.0% vs. −12.5±3.5%; *p*=0.018 and +1.0±6.4% vs. +3.5±6.4%; *p*=0.003 respectively). For most strain-based discoordination parameters, the SLICE method tended to produce slightly lower values compared to the CMR-TAG method (Tables 2 and 3).

**Table 2:** Basic strain parameters, dyssynchrony and discoordination by SLICE and CMR-TAG

	Parameter N = 27	SLICE	CMR-TAG	Mean difference	R^2^
Basic strain parameters	Systolic strain septum (%)	+1.0 ± 6.4	+3.5 ± 6.4	-2.5 ± 3.9	0.66
	Systolic strain rate septum (%/s)	+2.3 ± 18.1	+8.9 ± 17.9	-6.6 ± 10.1	0.68
	Diastolic strain rate septum (%/s)	+1.3 ± 14.6	-2.4 ± 12.2	+3.7 ± 10.8	0.48
	Systolic strain lateral wall (%)	-13.9 ± 4.0	-12.5 ± 3.5	-1.4 ± 2.9	0.50
	Systolic strain rate lateral wall (%/s)	-36.4 ± 10.9	-32.8 ± 9.4	-3.6 ± 7.7	0.53
	Diastolic strain rate lateral wall (%/s)	-32.9 ± 15.4	-27.9 ± 12.2	-5.0 ± 8.4	0.70
Dyssynchrony	Onset-delay (ms)	75 ± 34	58 ± 5	+17 ± 29	0.29
	Peak-delay (ms)	241 ± 130	239 ± 114	+2 ± 121	0.27
Discoordination	SF (%)	1.9 ± 2.1	1.9 ± 1.8	0.0 ± 1.5	0.51
	SRS (%)	6.3 ± 3.9	8.5 ± 5.2	-2.3 ± 3.0	0.66
	SSI (%)	7.9 ± 4.7	9.9 ± 6.0	-1.9 ± 3.2	0.73
	ISF	0.38 ± .23	0.51 ± .28	-0.13 ± 0.20	0.48

*SLICE* segment length in cine, *CMR-TAG* myocardial tagging, *onset-delay* septum to lateral delay onset contraction, *peak-delay* time difference in peak shortening between septum and lateral wall, *SF* septal flash, *SRS* systolic rebound stretch of the septum, *SSI* systolic stretch index, *ISF* internal stretch factor

**Table 3:** Detection of differences in strain parameters between ICMP and NICMP patients

	Parameter N = 27	SLICE			CMR-TAG		
		ICMP	NICMP	*p*-value	ICMP	NICMP	*p*-value
Basic strain parameters	Systolic strain septum (%)	-7.4	+4.0	<0.001	-4.1	+6.2	<0.001
	Systolic strain rate septum (%/s)	-21.3	+10.6	<0.001	-12.4	+16.4	<0.001
	Diastolic strain rate septum (%/s)	+18.3	-4.6	<0.001	+12.2	-7.4	<0.001
	Systolic strain lateral wall (%)	-9.7	-15.3	<0.001	-8.9	-13.7	0.030
	Systolic strain rate lateral wall (%/s)	-26.8	-39.7	0.005	-23.5	-36.0	0.001
	Diastolic strain rate lateral wall (%/s)	+16.3	+38.8	<0.001	+15.2	+32.4	<0.001
Dyssynchrony	Onset-delay (ms)	81	73	0.713	41	63	0.166
	Peak-delay (ms)	108	287	0.001	135	275	<0.001
Discoordination	SF (%)	0.9	2.3	0.147	0.8	2.3	0.050
	SRS (%)	2.4	7.6	0.001	4.5	9.9	0.014
	SSI (%)	4.4	9.2	0.017	5.1	11.5	0.012
	ISF	0.18	0.46	0.004	0.26	0.60	0.002

*SLICE* segment length in cine, *CMR-TAG* myocardial tagging, *ICMP* ischaemic cardiomyopathy, *NICMP* non-ischaemic cardiomyopathy, *onset-delay* septum to lateral delay onset contraction, *peak-delay* time difference in peak shortening between septum and lateral wall, *SF* septal flash, *SRS* systolic rebound stretch of the septum, *SSI* systolic stretch index, *ISF* internal stretch factor

### Agreement SLICE and CMR-TAG

**Fig. 4:**
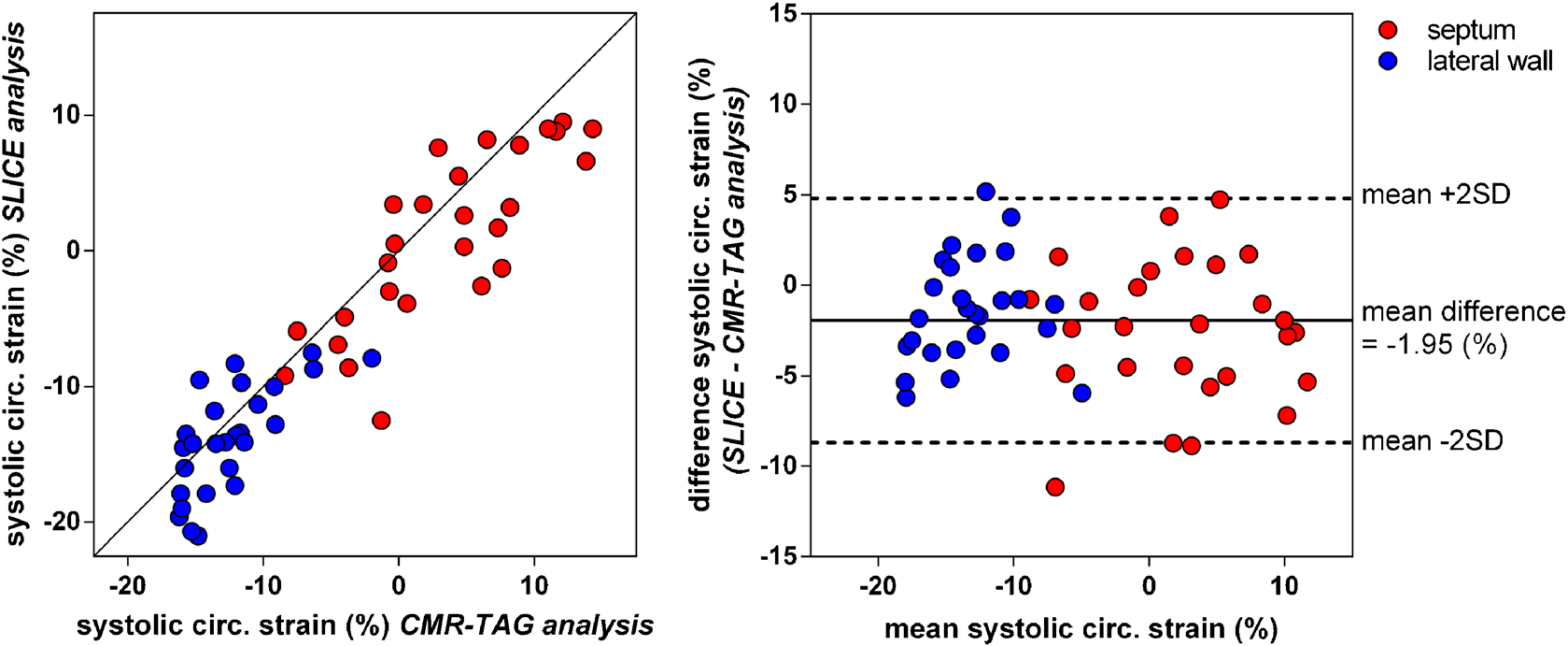
Agreement of segmental strains between the segment length in cine (SLICE) and the cardiovascular magnetic resonance – myocardial tissue tagging (CMR-TAG) technique. The left panel shows the correlation between systolic strains measured by CMR-TAG and SLICE. Corresponding Bland-Altman analysis (right panel) shows close agreement between the CMR-TAG and SLICE technique with the SLICE analysis resulting in slightly more negative strain values compared to the CMR-TAG analysis

Systolic strain values of the septum and the lateral wall showed close agreement between the SLICE and CMR-TAG technique, as illustrated in Fig. 4. Bland-Altman analysis affirms agreement between both methods, with slightly more negative strain values for the SLICE analysis compared to the CMR-TAG analysis (mean difference: −1.95±6.8%). Measures of absolute agreement for segmental strains, strain rates and indices of dyssynchrony and discoordination are summarized in Table 4. Overall, discoordination parameters demonstrated good agreement between both methods, whereas measures of mechanical dyssynchrony showed moderate agreement. Classification of septal strain patterns showed good agreement between both modalities, as presented in Table 5.

**Table 4:** Intraclass correlation coefficient for absolute agreement (ICC) and 95% CI

		SLICE – CMR-TAG N = 27	SLICE intra-observer N = 15	SLICE interobserver N = 15
Basic strain parameters	Systolic strain septum (%)	ICC 0.76 (0.43–0.90)	ICC 0.94 (0.82–0.98)	ICC 0.86 (0.63–0.95)
	Systolic strain rate septum (%/s)	ICC 0.78 (0.47–0.91)	ICC 0.94 (0.82–0.98)	ICC 0.86 (0.63–0.95)
	Diastolic strain rate septum (%/s)	ICC 0.66 (0.39–0.83)	ICC 0.93 (0.79–0.98)	ICC 0.87 (0.67–0.96)
	Systolic strain lateral wall (%)	ICC 0.66 (0.35–0.83)	ICC 0.77 (0.36–0.92)	ICC 0.67 (−0.05–0.90)
	Systolic strain rate lateral wall (%/s)	ICC 0.68 (0.39–0.85)	ICC 0.76 (0.35–0.92)	ICC 0.61 (−0.05–0.87)
	Diastolic strain rate lateral wall (%/s)	ICC 0.77 (0.47–0.90)	ICC 0.91 (0.71–0.97)	ICC 0.74 (0.06–0.92)
Dyssynchrony	Onset-delay (ms)	ICC 0.45 (0.09–0.71)	ICC −0.21 (−0.54–0.27)	ICC 0.20 (−0.36–0.64)
	Peak-delay (ms)	ICC 0.52 (0.17–0.57	ICC 0.74 (0.39–0.91)	ICC 0.37 (−0.15–0.73)
Discoordination	SF (%)	ICC 0.71 (0.46–0.86)	ICC 0.72 (0.34–0.90)	ICC 0.36 (−0.18–0.73)
	SRS (%)	ICC 0.70 (0.27–0.87)	ICC 0.83 (0.57–0.94)	ICC 0.73 (0.37–0.90)
	SSI (%)	ICC 0.78 (0.49–0.90)	ICC 0.81 (0.49–0.93)	ICC 0.72 (0.35–0.89)
	ISF	ICC 0.61 (0.22–0.81)	ICC 0.91 (0.74–0.97)	ICC 0.61 (0.16–0.85)

*SLICE* segment length in cine, *CMR-TAG* myocardial tagging, *onset-delay* septum to lateral delay onset contraction, *peak-delay* time difference peak shortening between septum and lateral wall, *SF* septal flash, *SRS* systolic rebound stretch of the septum, *ISF* internal stretch factor

**Table 5:** Cross table with LBBB patterns between SLICE and CMR-TAG SLICE intra-observer and SLICE interobserver

		CMR-TAG N = 27			SLICE intra-observer N = 15			SLICE interobserver N = 15		
		LBBB-1 n (%)	LBBB-2 n (%)	LBBB-3 n (%)	LBBB-1 n (%)	LBBB-2 n (%)	LBBB-3 n (%)	LBBB-1 n (%)	LBBB-2 n (%)	LBBB-3 n (%)
SLICE	LBBB-1 n (%)	5 (19)	2 (7)	0 (0)	1 (7)	1 (7)	1 (7)	2 (13)	0 (0)	1 (7)
	LBBB-2 n (%)	2 (7)	11 (41)	0 (0)	1 (7)	8 (53)	0 (0)	1 (7)	8 (53)	0 (0)
	LBBB-3 n (%)	1 (4)	0 (0)	6 (22)	0 (0)	0 (0)	3 (20)	0 (0)	0 (0)	3 (20)
		Total agreement: 81%			Total agreement: 80%			Total agreement: 87%		
		Kappa value: 0.71 (*p* <0.001)			Kappa value: 0.64 (*p* =0.001)			Kappa value: 0.77 (*p* <0.001)		

*SLICE* segment length in cine, *CMR-TAG* myocardial tagging, *LBBB-1* left bundle branch block pattern 1, *LBBB-2* left bundle branch block pattern 2, *LBBB-3* left bundle branch block pattern 3

### Reproducibility of SLICE

For intra-observer variability assessment, SLICE analysis was repeated by the primary investigator in a subset of 15 patients, and showed excellent agreement between segmental systolic strains as displayed in Table 4. Intra-observer variability for all other strain-based discoordination parameters also proved to be excellent, with the exception of SF (good agreement). Intra-observer agreement for indices of mechanical dyssynchrony was good for peak-delay, but poor for onset-delay. Intra-observer agreement for the classification of septum strain patterns was good. Additionally, a second observer independently analysed the same subset of patients to determine the interobserver variability, which was excellent for segmental systolic strains in the septum and good for the lateral wall. Interobserver variability for parameters of discoordination and mechanical dyssynchrony were found to be good for all discoordination parameters, with the exception of SF (poor agreement), and poor for parameters of mechanical dyssynchrony. Interobserver agreement was good for the classification of septal strain patterns.

### Detection of mechanical discoordination

**Fig. 5:**
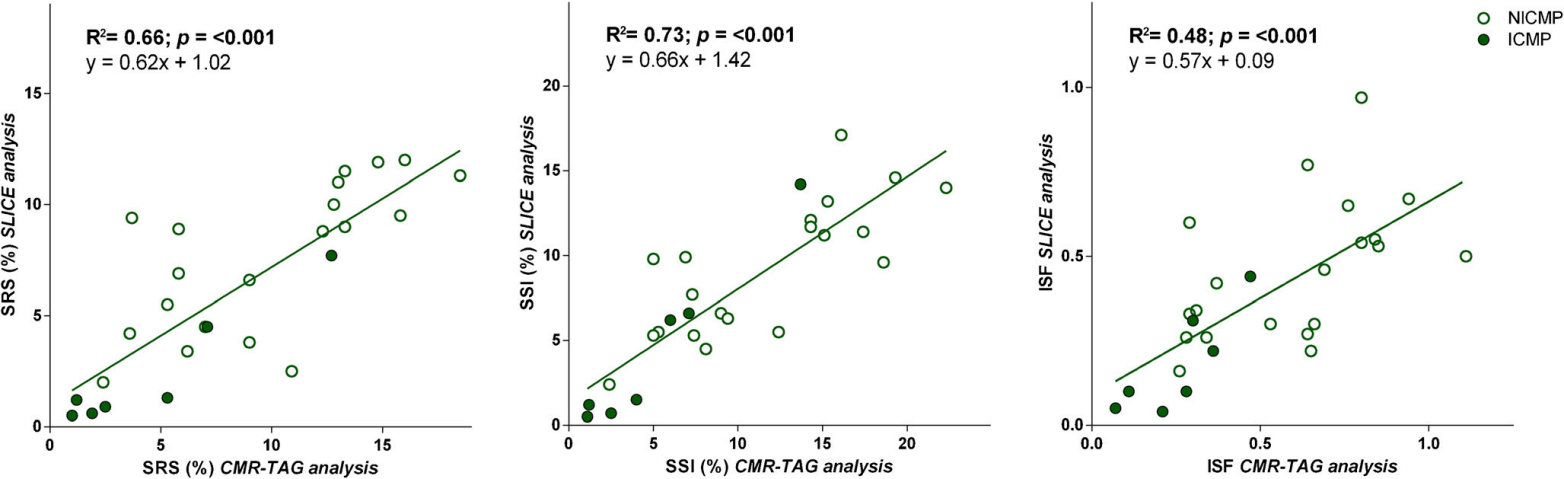
Agreement of predictors of Cardiac Resynchronization Therapy (CRT) response between the segment length in cine (SLICE) and cardiovascular magnetic resonance – myocardial tissue tagging (CMR-TAG) technique. There is a strong correlation between SLICE and CMR-TAG-derived strain measures with respect to the commonly used predictors of CRT response being systolic rebound stretch of the septum (SRS; left panel), systolic stretch index (SSI; middle panel) and internal stretch factor (ISF; right panel)

Mechanical discoordination was assessed by calculating the commonly used parameters SRS, SSI and ISF [5, 6, 23]. Figure 5 illustrates these parameters assessed by the SLICE method and the CMR-TAG method to be strongly correlated. Using the ‘reference’ CMR-TAG technique, significant differences in discoordination values were detected between ischaemic cardiomyopathy (ICMP) and non-ischaemic (NICMP) patients with the ICMP group showing lower rates compared to NICMP patients (SRS 4.5±4.3% vs. 9.9±4.8%; *p*=0.014; SSI 5.1±4.4% vs. 11.5±5.7%; *p*=0.012; ISF 0.26±0.14 vs. 0.60±0.26; *p*=0.002). Using the SLICE technique similar differences were detected between the ICMP-group and the NICMP-group (SRS 2.4±2.7% vs. 7.6±3.3%; *p*=0.001; SSI 4.4±5.0% vs. 9.2±4.0%; *p*=0.017; ISF 0.18±0.15 vs. 0.46±0.21; *p*=0.004); see Table 3.

## Discussion

This study demonstrates that the SLICE post-processing technique on standard CMR cine images offers both accurate and robust circumferential strain measures compared to the gold standard CMR-TAG technique in CRT candidates. Strain measures on a segmental scale, particularly of interest in this patient group to predict CRT response, show close agreement with the CMR-TAG technique, whereas recently published data on CMR-FT software showed disappointing results [13–17].

Chronic HF patients with LBBB, eligible for CRT, show large diversity in the manifestation of mechanical timing differences (dyssynchrony) and deformation abnormalities (discoordination) throughout the LV. Traditionally, echocardiographic modalities offer high temporal resolution imaging, which enables the assessment of regional timing differences throughout the LV. However, echocardiographic dyssynchrony parameters showed disappointing results in the PROSPECT study [8]. More recent, single-centre studies showed mechanical discoordination rather than mechanical dyssynchrony to be predictive for CRT response [4–7]. In this study, quantification of mechanical discoordination showed wide variation among CRT candidates, ranging from a normally coordinated to a severely discoordinated LV contraction pattern, with predominant stretching of the septum during systole (LBBB-2 pattern). Because of this wide interperson variation in segmental strains and discoordination rates, segmental strain analysis holds the potential to discriminate between CRT responders and non-responders at baseline. Using SLICE, the presence of a paradoxical septum movement was distinctly noticeable by qualitative assessment of the SLICE strain-curve (Fig. 1a). Classification of septum strains to pre-specified strain patterns (Fig. 3) showed good agreement between the SLICE and CMR-TAG method. Classification of a pseudonormal (LBBB-3) septum strain pattern is of clinical relevance as this pattern is associated with smaller benefit of CRT compared to other septum strain patterns [25]. Quantification of strain patterns by measuring regional systolic strains, the cumulative amount of SRS, SSI and the discoordination rate ISF showed good to excellent agreement with the CMR-TAG technique (Figs. 4 and 5). These are conventional markers of mechanical discoordination that have been shown to be accurate in the prediction of CRT response in multiple single centre studies [4–6, 23]. In addition, sensitivity of the SLICE technique proved to be high enough to detect the differences in discoordination values that were found between IMCP patients and NICMP patients. Patients with an ICMP have significantly lower discoordination values compared to NICMP patients, which might explain the lack of CRT response in this subgroup of patients. In general, the SLICE technique tends to produce slightly lower discoordination values compared to the CMR-TAG standard. This might be due to the lower temporal resolution that was used for cine imaging compared to CMR-TAG (~50 ms vs. ~15 ms) being less sensitive for peak strain values [28]. It is possible that this shortcoming in temporal resolution had a larger effect on the assessment of time-based dyssynchrony parameters, which could explain the lack of agreement and reproducibility that was found for these parameters. However, dyssynchrony parameters have limited predictive value for the selection of CRT patients [8]. Therefore, future studies should focus on the classification of septal strain patterns and discoordination markers, which can be accurately derived by the SLICE post-processing technique. Also, accelerated CMR imaging techniques such as parallel imaging can overcome this problem by improving the spatiotemporal resolution of the cine image acquisition [29]. However, since the SLICE technique is intended as a post-processing technique on standard cine images, a temporal resolution of ~50 ms was used for the acquisition of cine images as this is typical in most standard clinical protocols.

Conceptual differences between the SLICE and CMR-TAG techniques should be considered. CMR-TAG sequences cover the LV with magnetization saturated bands in a grid format at end-diastole [30]. Post-processing of these ‘tagged’ images by tracing the displacement of the taglines throughout the cardiac cycle produces high quality measures of intramural shortening. On the other hand, SLICE is performed by measuring total segment length between two anatomical landmarks throughout all phases on standard cine images. This method produces a measure of relative frame-to-frame segment length change that, in essence, approximates the net result of all intramural strains combined throughout the segment. A disadvantage of the SLICE method is that it derives strain measures from the apparent in-plane motion of the anatomical landmarks. However, this apparent in-plane movement may also be caused by through-plane displacements of oblique or tapering structures that form the anatomical landmarks. For this reason we could only analyse the mid-LV slice, since this plane is relatively motion independent. CMR-FT software neglects these pitfalls and the user is not able to track and trace the analysis steps. This might be the reason for the previous failure of segmental strain analysis by the CMR-FT software. Another consequential disadvantage is the inability to determine LV torsion, as this analysis requires both basal and apical rotation measurements.

To our knowledge, this is the first study to demonstrate a post-processing technique on standard CMR cine images that offers both accurate and robust circumferential strain measures on a segmental scale in CRT candidates. Both the visual classification of septal strain patterns and the quantification of conventional discoordination parameters being SRS, SSI and ISF showed close agreement with the ‘gold standard’ CMR-TAG technique, bringing the use of accurate predictors of CRT response a step closer to clinical practice.

Some limitations need to be addressed. Firstly, the present study was specified to HF patients with LBBB only. However, this might also be considered a strength of this study since segmental strains are particularly of interest in this specific population. Secondly, some geographic selection bias may have occurred as only study participants that were included close to our centre were invited to participate in the present CMR sub-study. Furthermore, the processing time for the CMR-TAG analysis was less than 20 min compared to a maximum of 60 min for the SLICE method. Although the SLICE technique was more time-consuming in the present study, previously used CMR-TAG post-processing software techniques also proved to be time-consuming and less user-friendly compared to the new SinMod technique by *inTag*.

For future studies, the SLICE processing time can be substantially reduced (halved) by limiting the analysis to the systolic phase, since diastolic strains are not incorporated in discoordination markers (Fig. 2). In fact, segmental systolic strains can already be calculated after analysing two frames providing both end-diastolic and end-systolic segment length (Fig. 1a). The number of frames that are required per strain parameter is displayed in Fig. S2 in the Supplemental Material. Further reduction of processing time, as well as improvement of reproducibility and clinical applicability, can be achieved by automated assessment. Additionally, implementing radial taglines to standard cine imaging will facilitate the detection of the landmarks, thus further enhancing strain analysis. Ultimately, SLICE might serve as a new principle for integration in CMR-FT algorithms.

In conclusion, the novel SLICE post-processing technique requires standard cine images only and provides both accurate and robust myocardial strain measures on a segmental scale in HF patients with LBBB. Future studies will focus on the prognostic value of these strain measures in CRT candidates.

## Electronic supplementary material

Below is the link to the electronic supplementary material.

ESM 1 (DOC 1292 kb)

## Abbreviations

CMR: Cardiovascular magnetic resonance
CMR-FT: Cardiovascular magnetic resonance – feature tracking
CMR-TAG: Cardiovascular magnetic resonance – myocardial tissue tagging
CRT: Cardiac resynchronization therapy
CSPAMM: Complementary spatial modulation of magnetization
HF: Heart failure
ICC: Intraclass correlation coefficient
ICMP: Ischaemic cardiomyopathy
ISF: Internal stretch factor
LBBB: Left bundle branch block
LV: Left ventricular
MARC: Markers and response to CRT
NICMP: Non-ischaemic cardiomyopathy
SF: Septal flash
SLICE: Segment length in cine
SSFP: Steady-state free-precession
SSI: Systolic stretch index
SRS: Systolic rebound stretch

## References

[1] Kalam K, Otahal P, Marwick TH (2014). Prognostic implications of global LV dysfunction: a systematic review and meta-analysis of global longitudinal strain and ejection fraction. Heart.

[2] Gjesdal O, Remme EW, Opdahl A (2011). Mechanisms of abnormal systolic motion of the interventricular septum during left bundle-branch block. Circ Cardiovasc Imaging.

[3] Leenders GE, Lumens J, Cramer MJ (2012). Septal deformation patterns delineate mechanical dyssynchrony and regional differences in contractility analysis of patient data using a computer model. Circ Heart Fail.

[4] Prinzen FW, Vernooy K, De Boeck BW, Delhaas T (2011). Mechano-energetics of the asynchronous and resynchronized heart. Heart Fail Rev.

[5] De Boeck BWL, Teske AJ, Meine M (2009). Septal rebound stretch reflects the functional substrate to cardiac resynchronization therapy and predicts volumetric and neurohormonal response. Eur J Heart Fail.

[6] Kirn B, Jansen A, Bracke F, van Gelder B, Arts T, Prinzen FW (2008). Mechanical discoordination rather than dyssynchrony predicts reverse remodeling upon cardiac resynchronization. Am J Phys Heart Circ Phys.

[7] Risum N, Tayal B, Hansen TF (2015). Identification of typical left bundle branch block contraction by strain echocardiography is additive to electrocardiography in prediction of long-term outcome after cardiac resynchronization therapy. J Am Coll Cardiol.

[8] Chung ES, Leon AR, Tavazzi L (2008). Results of the predictors of response to CRT (PROSPECT) trial. Circulation.

[9] Kapetanakis S, Kearney MT, Siva A, Gall N, Cooklin M, Monaghan MJ (2005). Real-time three-dimensional echocardiography: a novel technique to quantify global left ventricular mechanical dyssynchrony. Circulation.

[10] Raman SV, Simonetti OP (2009). The CMR examination in heart failure. Heart Fail Clin.

[11] Oyenuga OA, Onishi T, Gorcsan J (2011). A practical approach to imaging dyssynchrony for cardiac resynchronization therapy. Heart Fail Rev.

[12] Claus P, Omar AM, Pedrizzetti G, Sengupta PP, Nagel E (2015). Tissue tracking technology for assessing cardiac mechanics: principles, normal values, and clinical applications. J Am Coll Cardiol Img.

[13] Augustine D, Lewandowski AJ, Lazdam M (2013). Global and regional left ventricular myocardial deformation measures by magnetic resonance feature tracking in healthy volunteers: comparison with tagging and relevance of gender. J Cardiovasc Magn Reson.

[14] Harrild DM, Han Y, Geva T, Zhou J, Marcus E, Powell AJ (2012). Comparison of cardiac MRI tissue tracking and myocardial tagging for assessment of regional ventricular strain. Int J Cardiovasc Imaging.

[15] Morton G, Schuster A, Jogiya R, Kutty S, Beerbaum P, Nagel E (2012). Inter-study reproducibility of cardiovascular magnetic resonance myocardial feature tracking. J Cardiovasc Magn Reson.

[16] Singh A, Steadman CD, Khan JN (2015). Intertechnique agreement and interstudy reproducibility of strain and diastolic strain rate at 1.5 and 3 Tesla: a comparison of feature-tracking and tagging in patients with aortic stenosis. J Magn Reson Imaging.

[17] Wu L, Germans T, Guclu A, Heymans MW, Allaart CP, van Rossum AC (2014). Feature tracking compared with tissue tagging measurements of segmental strain by cardiovascular magnetic resonance. J Cardiovasc Magn Reson.

[18] Maass AH, Vernooy K, Wijers SC, et al (2017) Refining success of cardiac resynchronization therapy using a simple score predicting the amount of reverse ventricular remodelling: results from the Markers and Response to CRT (MARC) study. Europace. doi:10.1093/europace/euw445 10.1093/europace/euw445 28339818

[19] Zwanenburg JJ, Gotte MJ, Kuijer JP, Heethaar RM, van Rossum AC, Marcus JT (2004). Timing of cardiac contraction in humans mapped by high-temporal-resolution MRI tagging: early onset and late peak of shortening in lateral wall. Am J Physiol Heart Circ Physiol.

[20] Flett AS, Hasleton J, Cook C (2011). Evaluation of techniques for the quantification of myocardial scar of differing etiology using cardiac magnetic resonance. J Am Coll Cardiol Img.

[21] Schneider CA, Rasband WS, Eliceiri KW (2012). NIH Image to ImageJ: 25 years of image analysis. Nat Methods.

[22] Miller CA, Borg A, Clark D (2013). Comparison of local sine wave modeling with harmonic phase analysis for the assessment of myocardial strain. J Magn Reson Imaging.

[23] Lumens J, Tayal B, Walmsley J (2015). Differentiating electromechanical from non-electrical substrates of mechanical discoordination to identify responders to cardiac resynchronization therapy. Circ Cardiovasc Imaging.

[24] Marwick TH (2014). Selection for cardiac resynchronization therapy: all in a flash?. J Am Coll Cardiol Img.

[25] Leenders GE, Lumens J, Cramer MJ (2012). Septal deformation patterns delineate mechanical dyssynchrony and regional differences in contractility: analysis of patient data using a computer model. Circ Heart Fail.

[26] Castillo E, Osman NF, Rosen BD (2005). Quantitative assessment of regional myocardial function with MR-tagging in a multi-center study: interobserver and intraobserver agreement of fast strain analysis with Harmonic Phase (HARP) MRI. J Cardiovasc Magn Reson.

[27] Landis JR, Koch GG (1977). The measurement of observer agreement for categorical data. Biometrics.

[28] Lorsakul A, Gamarnik V, Duan Q (2012). Impact of temporal resolution on LV myocardial regional strain assessment with real-time 3D ultrasound. Conf Proc IEEE Eng Med Biol Soc.

[29] Kozerke S, Plein S (2008). Accelerated CMR using zonal, parallel and prior knowledge driven imaging methods. J Cardiovasc Magn Reson.

[30] Zerhouni EA, Parish DM, Rogers WJ, Yang A, Shapiro EP (1988). Human heart: tagging with MR imaging--a method for noninvasive assessment of myocardial motion. Radiology.

