# Supplementary material for: Strain analysis in CRT candidates using the novel segment length in cine (SLICE) post-processing technique on standard CMR cine images
Source: Eur Radiol. 2017 Jun 27;27(12):5158–68. doi: 10.1007/s00330-017-4890-0 (PMC5674110; doi:10.1007/s00330-017-4890-0)
Supplement: Supplementary file 1 — (DOC 1292 kb) [file 330_2017_4890_MOESM1_ESM.doc]

****SUPPLEMENTAL MATERIALS****

Strain analysis in CRT candidates using the novel segment length in cine (SLICE)

post-processing technique on standard CMR cine images

**SUPPLEMENTAL METHODS**

**Study population**

Inclusion criteria for the original MARC study were sinus rhythm, moderate to severe heart failure (New York Heart Association class II and III), intrinsic QRS-width ≥130ms with LBBB or ≥150ms without LBBB for patients with NYHA class II, intrinsic QRS-width ≥120ms with LBBB or ≥150ms without LBBB for patients with NYHA class III and at least 3 months stable optimal tolerated medical therapy. Patients with myocardial infarction within 40 days prior to study inclusion, coronary artery bypass graft or valve surgery within 90 days prior to study inclusion, previous pacemaker implantation or the presence of severe aortic stenosis were excluded.

**Image acquisition**

Typical image acquisition parameters: (1) cine imaging: slice thickness 5mm, slice gap 5mm, echo time (TE) 1.6ms, repetition time (TR) 3.2ms, temporal resolution <50ms, in-plane spatial resolution 1.5 by 2.1mm, flip angle 60 degrees. The number of reconstructed temporal phases within the cardiac cycle was set at 20; (2) high temporal cine imaging: TE 1.7ms, TR 3.4ms, temporal resolution ~15ms; (3) myocardial tagging: slice thickness 6mm, TE 1.7ms, TR 3.6ms, temporal resolution <15ms, in-plane spatial resolution 1.3 by 4.3mm, flip angle 20 degrees, tag spacing 7mm. The number of reconstructed temporal phases within the cardiac cycle was set at 55.

**Post-processing myocardial tissue tagging (CMR-TAG)**

After selecting the area of interest, endo- and epi-cardial contours were manually drawn in the end-systolic phase and automatically propagated (figure 1B). A template was placed dividing the LV in 6 equally sized regions (anterior, anterolateral, inferolateral, inferior, inferoseptal, anteroseptal). The myocardium was divided in 3 layers (endo-, mid-, epi-wall layer). Septum strain was calculated as the averaged strain from both the anteroseptal and inferoseptal segments of the mid-wall layer. Lateral wall strain was calculated as the averaged strain from both the anterolateral and inferolateral segments of the mid-wall layer. Individual strain curves were discarded in case of low signal-to-noise ratio as judged by two independent investigators. The presence of at least one analysable strain curve out of the two curves per segment resulted in inclusion of the patient for SLICE analysis.

**SUPPLEMENTAL FIGURE LEGENDS**

Figure S1: *Determination of the anatomical landmarks in the end-diastolic frame*

Step-by-step illustration to define anatomical landmarks in the end-diastolic mid-LV short-axis cine. First, a straight line was drawn from the anterior RV insertion point (marked as A) through the LV center point to locate the posterolateral region (marked as P) as shown in diagram (A). Subsequently, marks were placed perpendicular to the myocardium at the nearest trabecula that were traceable throughout the cardiac cycle, diagram (B). This procedure was repeated for the posterior RV insertion point (marked as P) and the anterolateral region (marked as A), diagram (C). Segment length was measured in *ImageJ* between both marks using a segmented line, diagram (D).

Figure S2: *Number of frames analysed per strain parameter*

Assessing basic strains (systolic strain, systolic strain rate and diastolic strain rate) requires only SLICE analysis of the end-diastolic (ED) and end-systolic (ES) frame. Obtaining dyssynchrony markers (onset-delay and peak-delay) requires SLICE processing of the septum and lateral wall in systolic frames. Discoordination markers can be calculated after analyzing septum SLICE in systolic frames (SF and SRS) or both the septum and lateral wall (SSI and ISF). Classification of septum strain patterns requires SLICE analysis of the septum in systolic frames. Red cross indicates septum SLICE analysis, blue cross indicates lateral wall SLICE analysis.

**SUPPLEMENTAL FIGURES**

Figure S1: *Determination of the anatomical landmarks in the end-diastolic frame*


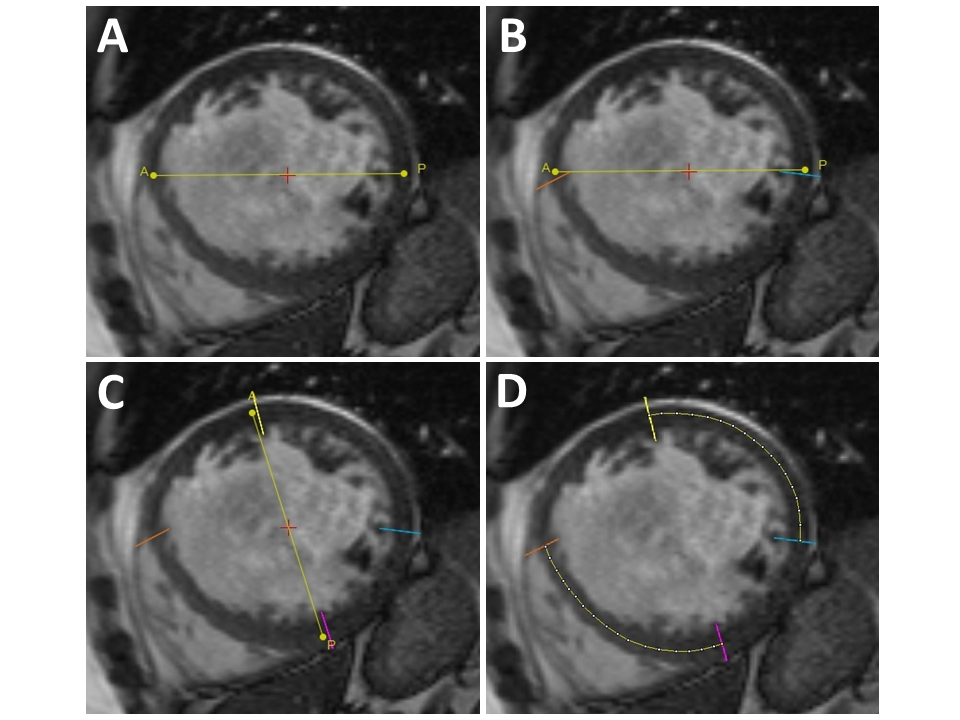


|  | | ED |  |  |  |  |  |  |  |  | ES |
| --- | --- | --- | --- | --- | --- | --- | --- | --- | --- | --- | --- |
| **Frames** | | **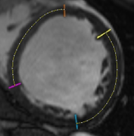**  **0 ms** | 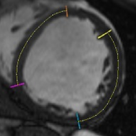  **40 ms** | 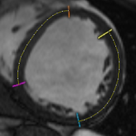  **81 ms** | 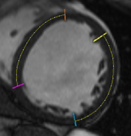  **121 ms** | 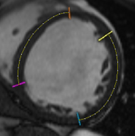  **162 ms** | 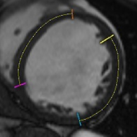  **202 ms** | 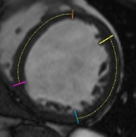  **243 ms** | 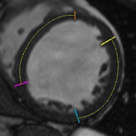  **283 ms** | 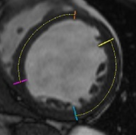  **324 ms** | 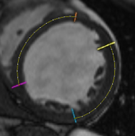  **364 ms** |
|  | |  |  |  |  |  |  |  |  |  |  |
| *basic strains* | systolic strain (/rate)  septum (%) |  |  |  |  |  |  |  |  |  |  |
|  | systolic strain (/rate)  lateral wall (%) |  |  |  |  |  |  |  |  |  |  |
|  |  |  |  |  |  |  |  |  |  |  |  |
| *dyssynchroy* | onset-delay (ms) |  |  |  |  |  |  |  |  |  |  |
|  | peak-delay (ms) |  |  |  |  |  |  |  |  |  |  |
|  |  |  |  |  |  |  |  |  |  |  |  |
| *discoordination* | SF (%) |  |  |  |  |  |  |  |  |  |  |
|  | SRS (%) |  |  |  |  |  |  |  |  |  |  |
|  | SSI (%) |  |  |  |  |  |  |  |  |  |  |
|  | ISF |  |  |  |  |  |  |  |  |  |  |
|  |  |  |  |  |  |  |  |  |  |  |  |
| septum patterns | |  |  |  |  |  |  |  |  |  |  |

Figure S2: *Number of frames analysed per strain parameter*
